# Characterization of mechanosensitive MSL gene family expression in *Zea mays* aerial and subterranean brace roots

**DOI:** 10.17912/micropub.biology.000759

**Published:** 2023-06-16

**Authors:** Olivia S Hazelwood, Ashley N Hostetler, Irene I Ikiriko, Erin E Sparks

**Affiliations:** 1 Department of Plant and Soil Sciences, University of Delaware, Newark, Delaware, United States

## Abstract

Plants must be able to sense and respond to mechanical stresses encountered throughout their lifespan. The MscS-Like (MSL) family of mechanosensitive ion channels is one mechanism to perceive mechanical stresses. In maize, brace roots emerge from stem nodes above the soil and some remain aerial while some grow into the soil. We tested the hypothesis that MSL gene expression is higher in subterranean brace roots compared to those that remain aerial. However, there was no difference in MSL expression between the two environments. This work sets the foundation for a deeper understanding of MSL gene expression and function in maize.

**Figure 1. MSL gene expression in maize brace roots varies by plant, but not environment (aerial or subterranean):**
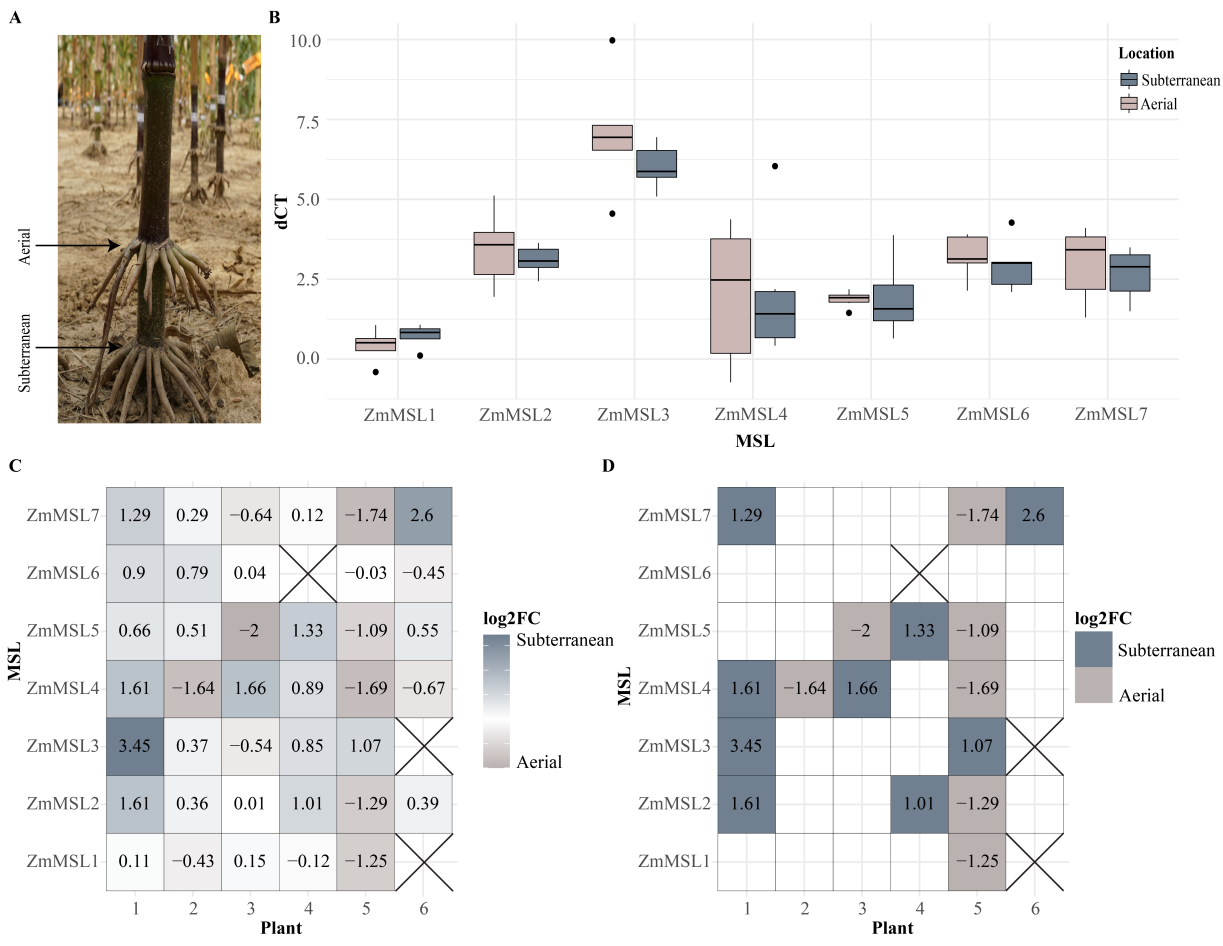
A) Maize brace roots are roots that develop from stem nodes above the soil. These roots may remain aerial (called aerial brace roots throughout) or enter the soil (called subterranean brace roots throughout). B) Analysis of quantitative Reverse Transcriptase (qRT-) PCR results shows that aerial and subterranean brace roots were not significantly different for MSL gene expression (p < 0.05). C) The log2 fold change expression of MSL genes between subterranean and aerial brace roots is shown for each plant. These log2 fold change values demonstrate the variation in expression differences between the two environments. D) Considering only expression values of log2 fold change > |1|, we see that the relative expression between root environments varies by plant.

## Description

Plants must perceive and respond to mechanical stimuli in order to survive. Examples of mechanical stimuli include external forces such as gravity, wind, and touch, as well as internal forces from cell division, cell expansion, and cell differentiation. These forces can often signal threats or significant developmental events, and in order for plants to respond to these stimuli, they must have mechanisms to detect it (Toyota et al. 2018) . Plants contain a number of conserved mechanosensitive (MS) ion channels that sense mechanical stress and can generate ion signals that facilitate the plant’s response to these mechanical stimuli (Basu and Haswell 2017; Guichard et al. 2022) . There are five families of MS proteins that have been discovered in plants and include the Mechanosensitive channel of Small conductance-Like (MSL), Hyperosmolality-gated Calcium-permeable channels (OSCA), Mid1-Complementing Activity (MCA), Piezo, and Two-Pore K ^+^ channel (TPK) (Guichard et al. 2022) . So far, the best studied of these MS channels are the MSL in the model plant *Arabidopsis thaliana* .

In Arabidopsis, six of the ten MSL (AtMSL) family proteins have been characterized. All of the characterized AtMSLs cluster in two distinct classes and act as osmotic safety valves, which function to relieve osmotic stress (Basu and Haswell 2017) . Class I proteins are organelle membrane localized and include the characterized AtMSL1-AtMSL3 proteins. AtMSL1 is important for modulating the bioenergetic function of the mitochondria (Lee et al. 2016) . AtMSL2 and AtMSL3 are localized to the plastid envelope and aid in maintaining plastid size and shape (Haswell and Meyerowitz 2006) . Class II proteins, which include AtMSL8-AtMSL10, are localized to the plasma membrane. AtMSL8 is expressed in the plasma membrane of pollen grains where it maintains cellular integrity required for pollen rehydration during fertilization (Hamilton et al. 2015) . Although a physiological role for AtMSL9 and AtMSL10 is yet to be identified, absence of stretch-gated activities in msl9-1;msl10-1 double mutants suggest that both proteins form a heteromeric complex in the plasma membrane of root cells during mechanosensing (Haswell et al. 2008; Peyronnet et al. 2008) . However, overexpression of AtMSL10 promotes cell death (Veley et al. 2014) and reduces susceptibility to *Pseudomonas syringae* (Basu et al. 2022) which suggests that these channels have, or may evolve to function in other roles. These foundational studies have provided groundwork for understanding the role of MSL proteins in sensing mechanical stimuli.

In contrast to Arabidopsis, the MS channels in many important crops such as *Zea mays* (maize) have been poorly characterized. Maize is an important cereal crop that is used to feed the world’s population and livestock, and in some countries contributes over 20% of food calories (Shiferaw et al. 2011) . However, maize yields are impacted by environmental forces such as wind and gravity that uproot the plants. Maize brace roots are a set of roots that grow from stem nodes above the soil and contribute to the anchorage of the stalk (Reneau et al. 2020) . These brace roots may remain aerial or grow into the soil and become subterranean ( **Figure 1A** ). However, there is nothing known about the function or pattern of expression of MSL genes in maize (ZmMSL) brace roots.

Generally, the expression of MS genes relative to their function is poorly defined. However, one study in *Mimosa pudica* showed that distal leaflet cells expressed lower density MS transcripts than proximal cells which are utilized in mechanotransduction, leading to folding of the leaflet (Tran et al. 2021) . Thus, suggesting that MS genes are expressed more highly in tissues that respond to mechanical stimuli. In this study, we hypothesized that ZmMSL gene expression would vary between aerial brace roots compared to subterranean brace roots. Specifically, we proposed that subterranean brace roots would experience greater mechanical stimuli and thus increase expression of the ZmMSL channels.

Maize has eight putative MSL genes (Kaur et al. 2020) . Similar to Arabidopsis, these ZmMSL genes can be divided into two classes: Class I (ZmMSL1-ZmMSL2) and Class II (ZmMSL3-ZmMSL8) (Kaur et al. 2020) . We designed primers to quantify the mRNA expression of all eight putative ZmMSL genes and designed reliable primers for seven of these. Thus, we quantified the expression of ZmMSL1-ZmMSL7 in aerial and subterranean brace roots for B73 inbred lines. Surprisingly, our results did not support our hypothesis and demonstrate that ZmMSL gene expression does not differ for aerial and subterranean brace roots ( **Figure 1B** ). In fact, some plants had increased expression for a specific ZmMSLs while others had decreased expression for the same gene. However, there is a difference in the expression among the putative ZmMSL genes, with ZmMSL3 being expressed the highest among the seven tested ZmMSL genes.

Using paired aerial and subterranean brace roots from each plant, we assessed the relative expression at the plant-level through log2 fold change analyses. We find that the differences in expression between aerial and subterranean brace roots is variable by plant ( **Figure 1C -D** ). This suggests that there may be a response of ZmMSL gene expression to external stimuli and environments, but these stimuli are occurring on the plant-level and remain to be fully elucidated. An alternative explanation is that the subterranean/aerial differences in ZmMSL expression at the plant-level may be due to differences in mechanical stresses that occurred during the sample collection (i.e. field excavation, washing, and root acquisition). However, this is unlikely given the short duration of sample collection (30 min). Further, we would have predicted that if differences in ZmMSL gene expression were due to sample preparation, then the subterranean roots would experience a greater mechanical stress given their contact with the soil and would in-turn have higher ZmMSL gene expression, but this was not the case. Future work repeating these experiments in a more controlled environment would help to resolve the potential impact of environment or sample collection. In addition, a detailed assessment of ZmMSL gene function is required to fully understand the role of these channels in maize growth, development, and environmental responsiveness.

## Methods

*Plant Material*

Maize seeds were planted in Newark, DE USA in the summer of 2019 as part of a larger field study. At 57 days after plant (approximately vegetative leaf 10 to 11 stage, V10-V11), six B73 plants were excavated from the field. A root ball of approximately 0.75 m in diameter was excavated with a shovel and placed in a 5-gallon bucket of water. Roots were washed with tap water to remove excess soil and one aerial and one subterranean brace root were collected from each plant, flash frozen in liquid nitrogen, and stored at -80℃ prior to analysis. Time between root ball excavation and brace root freezing was limited to 30 min or less to reduce the potential of inducing transcriptional responses.

*RNA Isolation and cDNA Synthesis*

Roots were ground with liquid nitrogen in a pre-cooled mortar and pestle. The Quick-RNA Plant Miniprep kit (Zymo Research) was used to extract RNA from ground root tissue. Approximately 50 mg of tissue was used in the protocol and samples were subjected to 4000 RPM for 2 min on the BeadBug homogenizer (Benchmark Scientific) prior to extraction. RNA concentration was assessed using a DS-11 FX Nano UV-Vis Spectrophotometer / Fluorometer (DeNovix). cDNA was synthesized from 1 ug of total RNA using the SuperScript VILO kit (ThermoFisher Scientific). Undiluted cDNA was used for qRT-PCR on a QuantStudio3 Real-Time PCR machine (ThermoFisher Scientific). Three technical replicates were run for each sample.

*Primer Design*

Primers targeting the eight ZmMSL genes were designed from the maize B73 reference genome v4. cDNA and genomic DNA sequences for ZmMSL1 through ZmMSL8 were retrieved from MaizeGDB (https://www.maizegdb.org/) (Woodhouse et al. 2020) . Splice junctions were manually annotated in ApE software. Primers were designed in Primer3Plus (Release 3.2.0; www.primer3plus.com) (Untergasser et al. 2020) to span splice junctions. Standard curves were run for each primer pair, and primers with an efficiency of 1.8 or greater were retained for analysis. We were unable to find reliable primers for ZmMSL8, thus only primers for ZmMSL1-ZmMSL7 were retained for analysis.

*qRT-PCR*

qRT-PCR was performed using SybrGreen chemistry with 20 uL reaction volumes (10 uL 2X Apex qPCR GREEN Master Mix; 8 uL water; 0.5 uL of 10 uM Forward; 0.5 uL of 10 uM Reverse; 1 uL cDNA) and three technical replicates. For standard curves, equal volumes of each cDNA sample were mixed to generate a 1X standard. This standard was then serially diluted with water to generate a 0.5X, 0.25X and 0.125X standard. Gene-specific reactions were run on the same plate as the housekeeping gene (ZmACT1). Reactions were run on a QuantStudio3 Real-Time PCR machine (ThermoFisher Scientific) with the following parameters: two holds of 50℃ for 20 min and 95℃ for 10 min, then 40 cycles of 95℃ for 15 sec and 60℃ for 1 min, followed by a melt-curve analysis.

*qRT-PCR Analyses*

Relative gene expression values for each of the seven ZmMSL genes (Table 1) were calculated via the 2 ^-ddCt^ method (Schmittgen and Livak 2008) . Threshold cycle (Ct) values were determined by the QuantStudio3 Real-Time PCR machine (ThermoFisher Scientific). Briefly, Ct values for technical replicates were averaged when the standard deviation was less than 0.50. The averaged Ct values of the technical replicates were then used to calculate the dCt value (dCt = Ct _GOI_ - CT _HK_ , where GOI is the gene of interest and HK is the housekeeping gene), where ZmACT1 was used as the housekeeping gene (Ding et al. 2020) and one of the seven ZmMSLs was used as the gene of interest. To calculate the ddCT value, aerial and subterranean root samples from the same plant (paired samples) were compared, and the dCt value for the aerial sample was subtracted from the dCt value for the subterranean sample (ddCt = dCt _Subterranean_ - dCT _Aerial_ ). Lastly, to calculate the fold change, 2 to the negative ddCt was calculated for each plant (Fold change = 2 ^-ddCt^ ). Fold change values were taken to log base 2 (Log2FC) for graphing.

*Statistical Analyses*

All statistical analysis and graphing were completed in R v.4.0.3 (R Core Team 2021) . Due to the nature of RT-qPCR data, a Kruskal-Wallis test (non-parametric equivalent to a one-way ANOVA) was used to determine if there was a significant difference between dCT values for aerial and subterranean brace roots. Individual Kruskal-Wallis tests were run for each of the seven ZmMSL genes. All figures were generated in R with the ggplot2 package ver. 3.3.3 (Wickham 2016) .

## Reagents

Table 1. Gene ID and primer sequences.

**Table:** 

Gene	Class	Gene Model (v3; v4)	Forward (5'-3')	Reverse (5'-3')
ZmMSL1	I	GRMZM2G028914;Zm00001d029608	TAACTGCTGGAGGGTTTGGG	TGCCAGAGACATCAACACCA
ZmMSL2	I	GRMZM2G125494; Zm00001d002580	GCTCTCTCTTTGGGCTACCG	ACATGACCGACGCCAAATTG
ZmMSL3	II	GRMZM2G027891; Zm00001d002679	GATCTTCCAGAACGTCGCCA	CCTCTTGCTGACCCTGTTGT
ZmMSL4	II	GRMZM2G303244; Zm00001d051415	TTCATGAGGCAGGAAGAGGC	AGCCATCTGGTTCAGCTTGT
ZmMSL5	II	GRMZM2G005996; Zm00001d017539	GAGGGGGATGAATCAGCGAC	GTGCTCCTGTGCTCCTTCAA
ZmMSL6	II	AY530952.1_FG001; Zm00001d037120	AGCAATCCGAAGCTCTAGGC	TCAGCAGATCCACCTCCTCA
ZmMSL7	II	GRMZM2G005013; Zm00001d044976	AGAGGTGGATCTGCTGAGGT	ACGAGGTTGTGAAGCTGCAT
ZmMSL8	II	GRMZM2G090627; Zm00001d026135	N/A	N/A
ZmACT1	HK	GRMZM2G126010; Zm00001eb348450	GCCCTGCTGTATGAAATGGA	AAAGGAACCAGCTAAAAGCAAAC
